# Editorial: Advances and applications of predictive toxicology in knowledge discovery, risk assessment, and drug development

**DOI:** 10.3389/fphar.2025.1721364

**Published:** 2025-10-20

**Authors:** Fengxian Zhang, Feiyu Li, Jiaqi Tan, Nan Zhang, Yonglong Han, Jiayu Liao, Huaqiang Zhai

**Affiliations:** ^1^ Standardization Research Center of Traditional Chinese Medicine Dispensing, School of Chinese Materia Medica, Beijing University of Chinese Medicine, Beijing, China; ^2^ Shanghai Sixth People’s Hospital Affiliated to Shanghai Jiao Tong University School of Medicine, Shanghai, China; ^3^ Department of Bioengineering, College of Engineering, Bourns College of Engineering, University of California at Riverside, Riverside, CA, United States

**Keywords:** predictive toxicology, mechanistic studies, drug toxicity, risk assessment, new drug development

## 1 Introduction

In the 20th century, toxicology made slow progress due to overreliance on animal-based assays, incompatible with 3R ethical guidelines while dealing with species-specific inaccuracies, excessive resource use, and sluggish workflows that delayed progress. Moreover, traditional *in vivo* animal tests often contained uncertainties, making it challenging to reliably project chemical toxicities in humans. These two problems—compounded by growing public opposition to animal experimentation—prompted innovation: the 1980s witnessed the rise of *in vitro* systems and computational toxicology, laying the foundation for predictive toxicology.

Driven by converging advances in AI, molecular biology, and data science, this field differs from conventional models of acute and chronic toxicity testing. It uses computational and AI-driven data mining to identify patterns, exploiting existing toxicological datasets to map structure-toxicity relationships, construct predictive models, and forecast potential toxicities of novel compounds.

This Research Topic features 12 contributions aimed at enabling efficient drug toxicity prediction and evaluation through deeper mechanistic understanding and high-throughput risk management—revealing predictive toxicology’s transformative potential in clarifying toxicity mechanisms, refining risk assessment, and accelerating safe therapeutic innovation. By integrating computational tools, AI, and *in vitro* techniques, these studies focus on developing knowledgebases including toxic ingredients, dose-time-toxicity correlations, structure-toxicity links, and clinical toxicity profiles. Such efforts provide comprehensive data foundations for risk forecasting of drug candidates and deliver systematic, quantitative model evaluations for clinical toxicity risk prediction.

## 2 Predictive modelling and mechanism exploration

Many studies have indicated advancements in applying computational frameworks, AI, and interdisciplinary technologies to drug toxicity prediction and assessment. Through approaches like AI modeling, multi-omics analysis, and integration of mechanistic information, research outcomes in predicting drug-induced toxicities, interpreting their mechanisms, and conducting risk evaluations emphasize the critical role of these tools. They enhance the accuracy of toxicity forecasts, deliver mechanistic insights, and optimize drug safety assessments. For example, Zhao et al. developed an artificial neural network (ANN) model to predict linezolid-induced thrombocytopenia, achieving 96.32% accuracy, which significantly exceeded traditional logistic regression. This highlights of ANN capacity to handle complex nonlinear relationships in toxicity data. Similarly, Roberts et al. combined *in silico* epitope mapping with *in vitro* HLA-binding assays to assess the immunogenicity risks of salmon calcitonin impurities, demonstrating how multi-algorithm approaches enhance prediction robustness. These studies prove AI as a key of next-generation toxicity screening.

Qu et al. further advanced genotoxicity assessment by quantifying γ-H2AX via mass spectrometry in HepG2 cells, a biomarker for DNA double-strand breaks. Their platform detected dose-dependent genotoxicity across 34 chemotherapy agents, with anthracyclines (e.g., doxorubicin) showing the strongest signals, which aligns with clinical cardiotoxicity profiles. These methodologies provide high-resolution mechanistic insights and prioritize compounds for further evaluation.

Rodríguez-Belenguer et al. proposed a novel methodology that integrats mechanistic information (molecular initiating events, MIEs, based on Adverse Outcome Pathways, AOPs) and toxicokinetic (TK) data. By combining multiple QSAR models describing simpler biological phenomena (low-level models, LLMs) and quantitative *in vitro*-to-*in vivo* extrapolation (QIVIVE) models, the sensitivity is enhanced, making it suitable for application to other complex toxicological endpoints (Rodríguez-Belenguer et al., 2024).

Organ toxicity mechanisms were dissected using multi-omics and pathway analysis. Tang et al. revealed that entrectinib induces neuronal apoptosis by suppressing THBS1 and inhibiting the PI3K-AKT/TGF-β pathways, offering therapeutic targets for neurotoxicity mitigation. In hepatotoxicity, Gao et al. linked *Psoralea corylifolia* extract to FXR/PPARα dysregulation in zebrafish, causing bile acid accumulation and lipid metabolism disruption. These findings emphasize the value of pathway-centric models in identifying intervention points. Cardiovascular safety was addressed by Morris et al., who modeled hemodynamic regulation in rats and dogs, incorporating 50 secondary pharmacology targets. Their framework simulated circadian rhythms and drug-induced perturbations (e.g., dopamine’s effects on sympathetic activity), providing a template for species-specific toxicity extrapolations.

The Research Topic of delayed immune-related adverse events (irAEs) was explored by Yang et al., who analyzed FAERS data to characterize irAEs occurring >1 year post-ICI initiation. Gastrointestinal and endocrine disorders (ROR_025_ = 10.50) emerged as high-risk late toxicities, urging long-term patient surveillance. This complements the work of Roberts et al. on immunogenicity, highlighting the need for predictive models of delayed T-cell responses.

## 3 Data-driven risk assessment and validation

Large-scale pharmacovigilance studies have illuminated clinical toxicity patterns. Wu et al. mined the FDA Adverse Event Reporting System (FAERS) to profile KRAS G12C inhibitors (sotorasib/adagrasib), associating sotorasib with hepatobiliary disorders and adagrasib with renal injuries. Similarly, Li et al. identified rosuvastatin-fenofibrate combination risks (e.g., gastrointestinal bleeding) using disproportionality analysis, advocating vigilant clinical monitoring.

Huang et al. leveraged the NHANES data to link osteoporosis to 34 medication ingredients (e.g., levothyroxine and omeprazole), revealing underrecognized drug-induced bone loss. These real-world analyses bridge preclinical predictions and clinical outcomes, thereby refining risk stratification. Zhang et al. established a “toxic component-traditional Chinese medicine-adverse reaction” database targeting traditional Chinese medicines containing toxic components (such as Aconitum alkaloids, mineral medicines, and Arisaema family herbs), identifying four major clinical risk factors: drug-related factors (containing cold and cool medicinal properties/allergenic components), medication-related factors (overdose/treatment duration), individual factors (allergic constitution/special populations), and regulatory factors (incomplete instructions). Gastrointestinal damage (50.8%), skin and appendage damage (33.6%), and allergic reactions (11.0%) were the most prominent adverse events, providing direct evidence for clinical risk stratification of toxic traditional Chinese medicines (Zhang et al., 2024).

Innovative *in vitro* and cross-species platforms have enhanced toxicity prediction. Ma et al. conducted a 90-day rat study of *Lithocarpus litseifolius* extract, establishing a no-observed-adverse-effect level (NOAEL) of 2,000 mg/kg/day and validating its safety for traditional medicine applications. Sung et al. introduced MDTR (Multi-Dimensional Transcriptomic Ruler), a knowledge-guided tool for quantifying liver toxicity via KEGG pathways in transcriptomic data. MDTR outperformed conventional metrics in detecting dose-dependent hepatotoxicity, as seen in LINCS database compounds.

These studies discover clinical toxicity patterns and identify potential risks through large-scale database analyses, while also enhancing toxicity prediction capabilities via innovative *in vitro* experiments and cross-species platforms. Collectively, such efforts provide solid support for drug safety assessment and clinical risk management.

## 4 New drug development

Developing new pharmaceuticals is a complex, costly task, often stopped by late-stage problems resulting from unanticipated toxic effects. Predictive toxicology addresses this challenge by identifying potential risks early in development process—ultimately reducing expenses and improving patient safety during the drug development. It achieves this by combining chemical analyses, molecular biological frameworks, mathematical algorithms, and computational models to explore links between environmental xenobiotic exposure and chemical-induced adverse outcomes. This integration provides supportive strategies for risk assessment across pharmaceuticals, chemicals and related products.

By combining computational modeling, AI, and high-throughput *in vitro* screening, researchers improve their ability to predict and reduce drug-related risks. This Research Topic compiles various studies that highlight these advanced technologies in toxicological research, offering valuable insights into the mechanisms underlying drug toxicity and facilitating the creation of safer therapeutic compounds.

## 5 Conclusion

The research featured in this Research Topic has collectively driven progress in predictive toxicology by demonstrating how novel computational and lab-based methodologies can improve our understanding and management of drug-induced toxicity. Distinguished by mechanistic, human-focused models that link chemical structures to biological outcomes, rather than relying on observational endpoints, predictive toxicology has transformed risk assessment for chemicals, pharmaceuticals, and consumer products, becoming a key element in the development and application of modern toxicology.

Consequently, it is essential that we stress raising awareness of predictive toxicology and strictly evaluating associated risks, as these aspects are the foundation for turning scientific advances into practical safety frameworks. Especially, incorporating long-term and acute toxicity evaluations into predictive toxicological processes improves the reliability of toxicity forecasts, ensuring a more complete understanding of potential risks in different exposure situations. These advancements, combined with a greater emphasis on risk awareness, promote the development of safer pharmaceuticals and chemicals while reducing dependence on animal testing, aligning with both ethical imperatives and scientific progress.

However, fully realizing the potential of predictive toxicology requires continued research to address current challenges, including data quality, model interpretability, and regulatory acceptance. Through continued innovation, strengthened partnerships between academia, industry, and regulatory bodies, and a strong focus on risk assessment and awareness, predictive toxicology is expected to play an even more important role in safeguarding human health and environmental safety.
